# Salvarsan in Syphilis

**Published:** 1912-02

**Authors:** 


SALVARSAN IN SYPHILIS: G. Frank Lydston of Chicago, *New York Med. Jour.* October 21, 1911. Lydston claims that it is unnecessary to use a very dilute solution of salvarsan for intravenous injection. The solution is made in 5—10 c.c. of salt solution alkalized and injected very slowly into the vein with a Luer syringe. The rapid flow of blood in the vein diluting the solution sufficiently provided that the injection be made very slowly.

